# Longitudinal trends in clinical characteristics and lung function of patients with severe asthma under treatment in Brazil

**DOI:** 10.1186/s12890-016-0302-5

**Published:** 2016-11-09

**Authors:** P. C. A. Almeida, E. V. Ponte, A. Souza-Machado, A. A. Cruz

**Affiliations:** 1 ProAR – Center of Excellence for Asthma of Federal University of Bahia (UFBA), Salvador, Brazil; 2 Faculdade de Medicina de Jundiaí, São Paulo, Brazil; 3 Institute for Health Sciences of UFBA and Coordinator of ProAR, Salvador, Brazil; 4 Center of Excellence for Asthma of UFBA, Salvador, Brazil

**Keywords:** Asthma, Pulmonary disease, Longitudinal studies, Spirometry

## Abstract

**Background:**

The structural changes of the respiratory system related to ageing determine lung function decline in healthy subjects after 25 years of age. An annual reduction of 25 ml in Forced Expiratory Volume in 1 s (FEV1) is expected. We aimed to describe the longitudinal lung function variation of subjects with severe asthma receiving appropriate treatment.

**Methods:**

Consecutive patients enrolled in a Brazilian reference clinic between 2003 and 2006 were invited to participate. The study participants were followed up for a median of 8 years, and were evaluated with spirometry in three distinct occasions (V0, V1 and V8), at least. At V0, upon enrollment, subjects with previous severe untreated asthma were evaluated by a specialist, had their health resource utilization in the last 12 months recorded, and performed spirometry. In V1, 1 year after V0, under proper management, subjects repeated the procedures and answered the Asthma Control Questionnaire (ACQ) and the Asthma Quality of Life Questionnaire (AQLQ). In the last study visit (V8), 7 years after V1, all patients underwent a pre and post-broncodilator (postBD) spirometry, skin prick test for aeroallergens, answered the ACQ and the AQLQ and had another interview with the specialist.

**Results:**

Two hundred thirty-four subjects were followed up between V0 and V8. A comparison between spirometries of V1 and V8, after the initial improvement has supposedly reached a plateau, shows that the FEV_1_ and FVC declined significantly both in absolute and percent of predicted values. FEV_1postBD_ did not change significantly between V0 and V1, but declined by −27.1 (−51.1–1.4) ml/yr between V1 and V8.

**Conclusions:**

Currently available treatment with a combination of inhaled corticosteroids and LABA may not be sufficient to prevent lung function decline in subjects with severe asthma.

## Background

Ageing is associated with reduced chest wall compliance, impaired respiratory muscle performance and decreased of lung elastic recoil. The structural alterations of the respiratory system related to ageing determine lung function changes of healthy subjects after 25 years old. An annual reduction of 25 ml in Forced Expiratory Volume in 1 s (FEV_1_) is expected in subjects after 25 years of age [1], rising to 38 ml in subjects above 65 years old [2].

Cohort studies of subjects with asthma demonstrated that the disease can accelerate lung function decline [3, 4]. A baseline FEV_1_, longer disease duration, ageing and greater FEV_1_ variability are related to lung function decline above the average, among subjects with asthma [5–7].

Longitudinal studies demonstrated that inhaled corticosteroids may reduce lung function decline among adults and children with uncontrolled asthma, especially if associated with Long Acting Beta 2 Agonist (LABA) [8–12]. However, most trials had a short follow-up period. One retrospective study of subjects with asthma diagnosed by physician [13] and a prospective study of subjects with self-reported asthma [14] aimed to evaluate the effect of inhaled corticosteroids on long-term trends of lung function. Both of these long-term observational studies indicated that inhaled corticosteroids reduce the loss in lung function of subjects with asthma, as compared to subjects not using inhaled corticosteroids. A retrospective study of subjects with severe asthma treated for 10 years indicated that the decline in FVC is more evident than FEV_1_, suggesting that small airway susceptibility may be the cause of rapid disease progression. Aging, exacerbations of asthma, and use of systemic corticosteroids were related to excess FVC decline [15].

However, there remains a gap in the knowledge about the effect of proper treatment with inhaled corticosteroids in the long-term trends of lung function of subjects with previous untreated severe asthma. It is unknown whether sustained treatment with high dose of inhaled corticosteroids can keep lung function decline of subjects with severe asthma within the physiological range. The aim of our study was to describe the longitudinal lung function variation of subjects with severe asthma receiving appropriate treatment with inhaled corticosteroids and LABA regularly (adherence ≥80 %).

## Methods

### Study population

Consecutive patients enrolled at the Program for Control of Asthma in Bahia (ProAR) between 2003 and 2006 were invited to participate. ProAR was established to assist subjects with previous untreated severe asthma from the Brazilian National Health System (SUS). This Program has three components: health care, building capacity and research. All subjects enrolled received multidisciplinary assistance, underwent spirometry, took part of an educational program and received free medication: a combination of an inhaled corticosteroids and a long acting β_2_ agonist (LABA), in addition to rescue salbutamol spray.

We included subjects with severe asthma according to the NIH-NHBLI Guidelines for the Diagnosis and Management of Asthma, 1997 [16] and GINA 2002 criteria [17]. In brief, they had asthma and any one of: (i) daily or continuous symptoms; (ii) activities limited daily (symptoms with minor efforts); (iii) nocturnal symptoms > 2 times a week; (iv) use of bronchodilators: ≥ 2 times a day; or (v) Peak Expiratory Flow (PEF) or FEV_1_: <60 % of predicted. Subjects were not receiving regular treatment until enrollment, their age was ≥ 12 years old. Subjects with a smoking history > 9 pack/years, current smoking, other lung diseases or other conditions that could possibly interfere in the evaluation of asthma, such as chronic obstructive pulmonary disease (COPD), tuberculosis, heart failure (HF) were not included.

A sample calculation was performed and the size of sample composed by 171 subjects it should be enough to achieve a power of 99.8 % to detect differences between two groups with differences in FEV_1_ at least of 5.5 %.

The diagnosis of asthma was audited and confirmed before the last study visit, by two independent specialists. The opinion of a third specialist was requested in case of disagreement about diagnosis or exclusion. For the validation of asthma diagnosis, we used the clinical history, previous lung function tests (to confirm variable airflow obstruction) and chest radiography (to exclude other lung diseases). All patients were evaluated for the presence of concomitant chronic rhinitis and other relevant comorbidities.

The Institutional Review Board of MCO – Federal University of Bahia, approved the study. All subjects or their legal guardians signed informed consent.

### Study design

This is a prospective real-life analysis of clinical and functional aspects of a cohort of subjects with previous untreated severe asthma [18].

The study participants were followed up for a median of 8 years, and were evaluated with spirometry in three distinct occasions, at least. Their three main study visits were titled Visit 0 (V0 – enrollment), Visit 1 (V1 – first year) and Visit 8 (V8 – final visit). The procedures of each visit were summarized in Table 1.

**Table 1:** Outline of procedures at each of the three main study visits

V0 – enrollment	V1 - 1 year later	V8 – 8 years later
Specialist evaluation with collection of information on health resource utilization in the last 12 months	Specialist evaluation	Specialist evaluation
Check for inclusion and non-inclusion criteria	Records of exacerbation history (emergency visits, hospitalizations and the use of systemic corticosteroids) in the last 12 months	Records of exacerbation history (emergency visits, hospitalizations and the use of systemic corticosteroids) in the last 12 months
Evaluation of rhinitis	Multidisciplinary approach to treatment and health education	Multidisciplinary approach to treatment and health education
Spirometry	Spirometry	Spirometry
Record of inhaled medications dispensation (at the beginning)	ACQ^a^ Questionnaire	ACQ^a^ Questionnaire
	AQLQ^b^ Questionnaire	AQLQ^b^ Questionnaire
		Skin prick test

Note: ^a^Asthma Control Questionnaire (ACQ) [24, 25], ^b^Asthma Quality of Life Questionnaire (AQLQ) [26, 27]

Patients had a consultation with a specialist quarterly for asthma control evaluation and adjustment of the treatment. They had a pharmacist appointment monthly for collecting the medication according to their prescription and underwent a subjective evaluation of adherence. The summary of activities developed by patients during the follow-up is in Table 2.

**Table 2:** Summary of patient’s activities during the follow-up

Activities quarterly	Activities monthly	Activities annualy
Nurse evaluation (anthropometric data; evaluation and orientation about correct management of medication) Specialist consultation (control of asthma evaluation - GINA; history of health resources use; adjustment of treatment: step-down or step-up doses by guidelines)	Pharmacist consultation (evaluation of adherence and correct use of the devices) Educational meetings (speeches about knowledge in asthma, its treatment, other diseases associated, action plan, environment management)	Spirometry Other general exams

Note: *GINA* global initiative of asthma

### Assessment tools

#### Social-demographic and clinical questionnaires

A standardized case report form with socio-demographic and clinical information was routinely filled out by the assistent specialist during the first study visit, including information on age, educational level, age of asthma onset, rescue medication use, emergence visit need, use of oral corticosteroid and need for hospitalization due to asthma in the last 12 months. The information on health resource utilization and use of oral corticosteroid was collected in the subsequent study visits as well.

Anthropometric measurements, weight and height were measured in fasting before the last spirometry, and the body mass index (BMI) was calculated. Subjects with BMI ≥ 30 were considered as obese.

#### Spirometry

Spirometries were performed before the morning dose of LABA avoiding use of rescue short acting beta 2 agonists. The last visit was scheduled when there was no history of exacerbation or acute respiratory infection in the preceding 4 weeks.

The tests were performed by a trained physiotherapist with certification by Brazilian Thoracic Society (SBPT), using a KOKO spirometer (PDS Instrumentation Inc., Louisville, CO, EUA), according to the American Thoracic Society [19] protocol, adopting specific normality standards for Brazilians [20]. The bronchodilator (Salbutamol spray 400mcg) reversibility was considered positive when FEV_1_ increased at least 200 ml and 12 % from pre-bronchodilator (preBD) values [21]. The choice of the best maneuver was performed manually, only when the test presented quality A or B [22].

In V0, 195 subjects performed spirometry, but 22 exams were interrupted immediately after the preBD maneuver because of uncomfortable symptoms. Further 39 preBD tests were not performed because patients presented an exacerbation or uncomfortable symptoms on the day scheduled for the test. In V1, 191 patients performed spirometry. Forty-three subjects could not perform the test because they had an exacerbation or acute respiratory infection, and seven patients did the preBD phase but could not finish the test. In the last visit (V8), all patients performed a complete spirometry with pre and post-broncodilator (postBD) phases. One hundred thirty-nine subjects performed preBD and postBD tests in V0, V1 and V8.

The period from V0 to V1 was used for stabilization of symptoms and lung function, with adjustment of the dose of inhaled corticosteroid for optimization of treatment.

#### Skin prick test

Immediate-type hypersensitivity skin prick tests were performed with 14 aeroallergen extracts, on the forearm. The antigens tested were *Alternaria alternata, Aspergillus flavus, Aspergillus niger, Aspergillus fumigatus, Cladosporium herbarum, Dermatophagoides pteronyssinus, Dermatophagoides farinae, German cockroach, American cockroach, Cat hair, Dog epithelium, Paspalum notatum, Cynodon dactilon.* (GREER® Labs, EUA) and *Blomia tropicalis* (FDA Allergenics, Brazil). As a negative control, we used saline solution and the positive control was histamine. The puncture sites were 2 cm apart. The test was considered positive when at least one antigen induced a reaction with a diameter ≥ 3 mm greater than the negative control [23].

#### Asthma evaluation

The ACQ_6_ questionnaire was applied to measure asthma symptoms. It comprises five questions about symptoms and 1 question about rescue medication. Scores ≥ 1.5 identify uncontrolled asthma among subjects of our programme [24, 25]. The AQLQ questionnaire was applied to evaluate asthma related quality of life. Higher scores indicate better quality of life. Score ranges from 0 to 7 [26, 27].

Asthma exacerbations were accounted for every 3 months, at the appointment with the physician. The use of oral corticosteroids, hospital admissions and emergency room visits from asthma were carefully noted.

### Statistical analysis

The descriptive variables were presented as central and dispersion tendency measurements: mean, median, standard deviation, interquartile range. The association between continuous variables were assessed by means of Wilcoxon or Friedman Tests, when comparing 2 or 3 visits, respectively. McNemar test was used to verify associations between categorical variables.

All subjects were evaluated on social, demographic and clinical characteristics, and the comparison between visits used all participants. The spirometric measures were analyzed according to number of subjects that performed preBD (162) and postBD (139) maneuvers in all study visits.

The data tabulation and analysis was performed using a software program *Statistical Package the Social Sciences* for *Windows*, 16.0 (SPSS for Windows; SPSS Inc., Chicago, IL, USA).

## Results

**Fig. 1:**
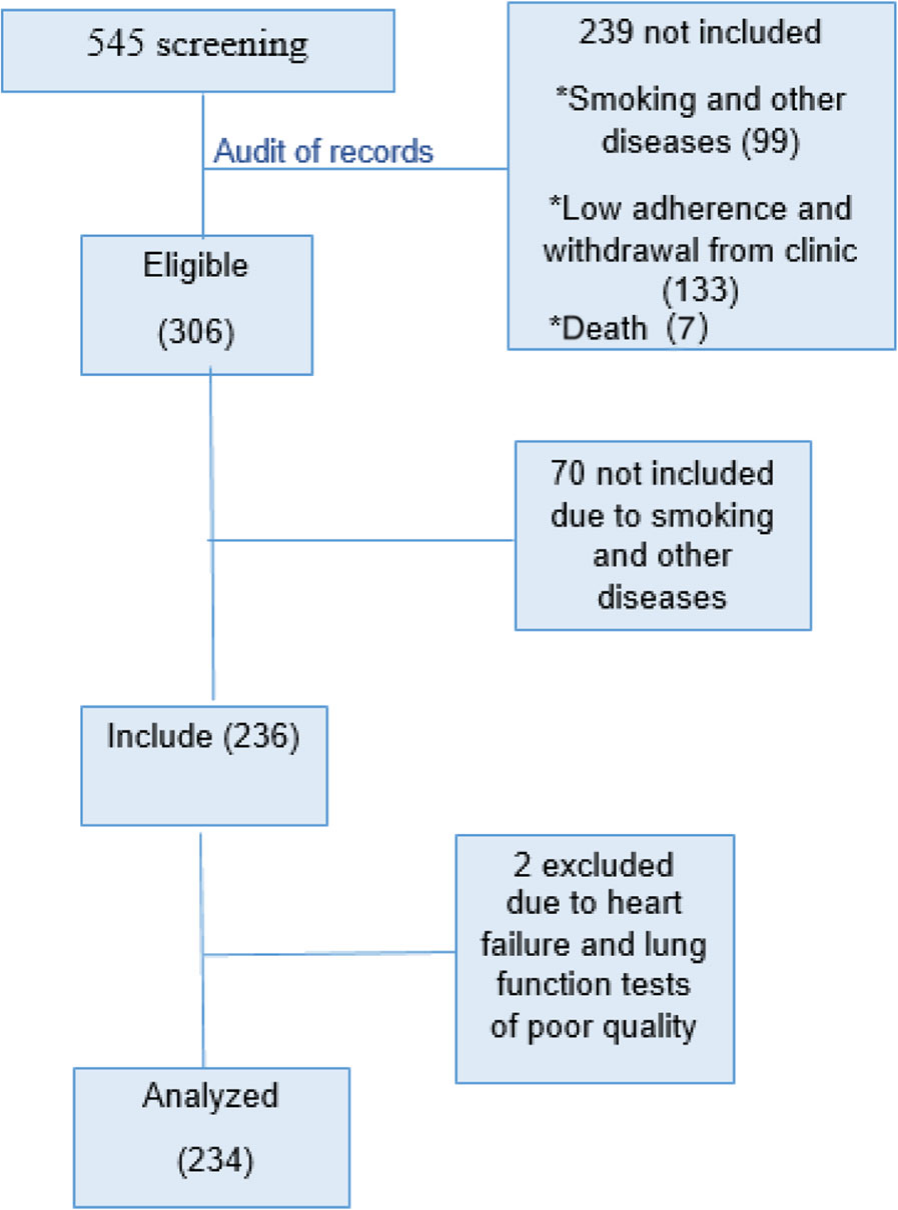
Flow diagram of study inclusion, non inclusion and exclusions. This flow diagram demonstrates the exclusions in the study during the follow-up

Over the period 2003–2006 we performed 545 screening, after audit of records we stayed on 306 eligible patients. We evaluated a total of 236 subjects in the study, however 2 were excluded, one presenting congestive heart failure diagnosis in the last visit and another because she didn’t perform any spirometry with good quality (A or B). The final analysis was performed with 234 subjects and the inclusion and exclusion of participants was demonstrated on the flow diagram depicted in Fig. 1. In the enrollment visit (V0) 192 (82 %) subjects were female, with a median age of 45 years old, 53 (23 %) were obese, the majority [199 (85 %)] had associated rhinitis and 131 (56 %) had a positive skin prick test, at least to 1 allergen.

The median age of asthma onset and of time without regular treatment was 8 and 30 years, respectively. The frequency of use of oral corticosteroids and emergency room visits in the year before admission were (*N* = 152; 65 %) and (*N* = 175; 75 %), respectively, due to asthma exacerbations. Table 3 describes the characteristics of the study population upon enrollment (V0).

**Table 3:** Clinical characteristics of all subjects at enrollment (V0)

Characteristics	Results
Subjects *n*	234
Female gender *n(%)*	192 (82)
Literate patients *n(%)*	203 (87)
Age in years *M(p*_*25*_ *-p*_*75*_ *)*	45 (35–54)
BMI *M(p*_*25*_ *-p*_*75*_ *)*	27 (24–31)
BMI ≥ 30 *n(%)*	54 (23)
History of chronic rhinitis *n(%)*	175 (74)
Positive skin prick test to aeroallergens *n(%)*	131 (56)
Age of asthma onset (yrs) *M(p*_*25*_ *-p*_*75*_ *)*	8 (2–23)
Duration of asthma without treatment (yrs) *M(p*_*25*_ *-p*_*75*_ *)*	30 (18–40)
Any oral corticosteroid requirement in the year before *n(%)*	152 (65)
Emergency room visits due to asthma in the year before (n) *M(p*_*25*_ *-p*_*75*_ *)*	4 (2–15)
Proportion of patients that need emergency room visits due to asthma in the year before *n(%)*	175 (75)
Proportion of patients that need ICU admission due to asthma once *n(%)*	38 (16)
Proportion of patients that need intubation due to asthma *n(%)*	14 (8)

Note: *BMI* body mass index, *ICU* intensive care unit, *yrs* years, *n (%)* number (proportion), *M(p*_*25*_ *-p*_*75*_ *)* median and interquartile range

### Clinical assessment during the follow up

The 234 subjects were followed up between V0 and V8. At enrollment in ProAR, a regular inhaled treatment was started. In the end of the first year of follow up, the participants had used the combination of formoterol 24mcg/day and an inhaled corticosteroid in a mean dose equivalent of 813.3 (±247.5) mcg/day of budesonide.

The subjects were reassessed 12 months after starting the inhaled therapy (V1). We observed a reduction in the frequency of emergency room visits (−26 %) and the use of oral corticosteroids (−28 %). The comparison between V0 and V1 is presented in Table 4.

**Table 4:** Clinical characterization from Visit 0 to Visit 1 (all subjects, *n* = 234)

Characteristics	Visit 0	Visit 1	*p* ^a^ value
Daily dose of inhaled budesonide equivalent in the last 3 months (μ ± SD)	^b^	813.3 (±247.5)	-
Use of long acting B_2_ agonists associated to inhaled corticosteroids n(%)	^b^	200 (86)	-
Any oral corticosteroid requirement in the year before the visit n(%)	152 (65)	86 (37)	<0.01
Number of emergency room visits due to asthma in the year before M(p_25_-p_75_)	5 (2–15)	0 (0–2)	<0.01
Proportion of patients that need emergency room visits due to asthma in the year before n(%)	172 (74)	113 (48)	<0.01
Proportion of patients that need ICU admission due to asthma in the year before n(%)	19 (9)	2 (1)	<0.01

Note: ^a^McNemar and Wilcoxon Tests. ^b^Subjects were not using regular inhaled corticosteroids before Visit 1. *M(p*_*25*_ *-p*_*75*_ *)* median and interquartile range

The final assessment was performed in the end of eighth year of regular treatment (V8). There was an increase in the mean dose of inhaled corticosteroid (mean of 1295.5 ± 754.8mcg/day) of budesonide or equivalent and a further reduction of 17 % in a proportion of emergency visits during the last year of follow up. The comparison between V1 and V8 is presented in Table 5.

**Table 5:** Clinical changes from Visit 1 to Visit 8 (all subjects, *n* = 234)

Characteristics	Visit 1	Visit 8	*p* ^a^ value
Daily dose of inhaled budesonide equivalent (*μ ± SD)*	813.3(±247.5)	1,295.5(±754.8)	<0.01
Any oral corticosteroid requirement in the year before *n(%)*	86 (37)	146 (62)	<0.01
Number of emergency room visits due to asthma in the year before *M(p*_*25*_ *-p*_*75*_ *)*	0 (0–2)	0 (0–1)	<0.01
Proportion of patients that need emergency room visits due to asthma in the year before *n(%)*	113 (48)	73 (31)	<0.01
Proportion of patients that need ICU admission due to asthma in the year before *n(%)*	2 (1)	4 (2)	0.69
AQLQ scores *M(p*_*25*_ *-p*_*75*_ *)*	4 (3–5)	5 (3–6)	<0.01
ACQ scores *M(p*_*25*_ *-p*_*75*_ *)*	2 (1–3)	1 (0–2)	<0.01
Proportion of patients with ACQ score ≥ 1.5 *n(%)*	141 (60)	87 (37)	<0.01
Proportion of patients with ACQ score ≤ 0.75 *n(%)*	44 (19)	97 (42)	<0.01

Note: *ACQ* asthma control questionnaire, *AQLQ* asthma quality of life questionnaire. ^a^McNemar and Wilcoxon Test

### Airflow limitation and BD response at each visit

In V0, 173 complete spirometries were performed (pre and postBD). One hundred twenty subjects (69.4 %) presented a FEV_1postBD_ <80 % and a positive BD response - with improvement ≥ 12 % and 200 ml on FEV_1_. Seventy (40.5 %) of them had a FEV_1_/FVC ratio ≥ 0.7 after BD.

In V1, 184 complete tests were performed and assessed. FEV_1 postBD_ < 80 % was present in 114 (61.6 %) subjects. Eighty-two (44.3 %) had FEV_1_/FVC ratio ≥ 0.7 after BD, and 42 (42.7 %) presented a positive response to BD.

All participants repeated spirometry in the last visit (V8). One hundred seventy-one (73.1 %) presented FEV_1postBD_ <80 %. Eighty-eight (37.6 %) had a FEV_1_/FVC ratio ≥ 0.7 after BD and 85 (36.3 %) presented a positive response to BD.

### Comparison of lung function between visits

#### Trends in preBD spirometric parameters

One hundred sixty-two subjects were evaluated preBD in all three study visits. A statistically significant increment was observed in all spirometric parameters after the first year of regular treatment (from V0 to V1). However, a comparison between spirometric parameters on V1 and V8, 7 years later, demonstrates a statistically significant reduction of FEV_1_ and FVC. The preBD lung function parameters are depicted in Table 6.

**Table 6:** Trends in pre-bronchodilator (PreBD) spirometric parameters during follow up

Study visits	V0	V1	V8	*p* value^a^	*p* value^a^	*p* value^b^
	(162)	(162)	(162)	V0xV1	V1xV8	V0xV1xV8
FVC (L)	2.5 (2.1–3.2)	2.7 (2.2–3.3)	2.4 (1.9–3.0)	<0.01	<0.01	<0.00
FVC (% pred)	82.0 (65.0–94.0)	88.0 (72.0–101.0)	77.1 (67.1–88.0)	<0.01	<0.01	<0.00
FEV_1_ (L)	1.5 (1.2–2.0)	1.8 (1.3–2.2)	1.5 (1.1–2.0)	<0.01	<0.01	<0.00
FEV_1_ (%pred)	59.5 (45–75.8)	66.5 (54.0–82.3)	63.0 (49.4–73.4)	<0.01	<0.01	<0.00
FEV_1_/FVC	0.6 (0.5–0.7)	0.7 (0.5–0.7)	0.7 (0.6–0.7)	0.04	0.69	0.03
FEV_1_/FVC (%pred)	75.0 (63.8–89.3)	79.0 (64.8–88.0)	80.4 (70.1–88.8)	0.02	0.16	<0.00
FEF_25–75%_ (L/s)	0.5 (0.8–1.3)	0.9 (0.5–1.7)	0.8 (0.6–1.4)	<0.01	0.04	<0.01
FEF_25–75%_ (%pred)	27.0 (17.0–48.5)	33.0 (21.0–52.0)	34.5 (24.1–51.0)	<0.01	0.19	<0.00

Note: ^a^Wilcoxon and ^b^Friedman tests; *FVC* forced vital capacity, *FEV1* forced expiratory volume in 1 s, *FEF* forced expiratory flow; data presented as median and interquartile range

#### Trends in postBD spirometric parameters

A hundred thirty-nine subjects performed complete lung function tests (pre and postBD) in all three study visits. Comparing the measurements enrollment visit (V0) with the observations after 1 year of regular treatment (V1), there was a statistically significant increase only in FEF_25–75%_, whereas a marked reduction in the response to bronchodilator was demonstrated (Table 7).

**Table 7:** Trends in post-bronchodilator (PostBD) spirometric parameters during follow up

Study visits	V0	V1	V8	*p* value^a^	*p* value^a^	*p* value^b^
	(139)	(139)	(139)	V0xV1	V1x V8	V0xV1xV8
FVC L	2.7 (2.3–3.5)	2.8 (2.3–3.4)	2.6 (2.1–3.1)	0.98	<0.01	0.00
FVC (% pred)	91.0 (77.0–101.0)	91.0(79.0–102.0)	82.2 (73.6–92.0)	0.43	<0.01	0.00
FEV_1_ (L)	1.9 (1.4–2.3)	1.9 (1.5–2.4)	1.7 (1.3–2.2)	0.41	<0.01	0.00
FEV_1_ (%pred)	73.0 (60.0–85.0)	73.0 (63.0–86.0)	67.2 (56.3–80.3)	0.15	<0.01	0.00
FEV_1_/FVC	0.7 (0.6–0.8)	0.7 (0.6–0.8)	0.7 (0.6–0.7)	0.34	0.21	0.47
FEV_1_/FVC (%pred)	82.0 (68.0–93.0)	83.0 (71.0–92.0)	81.5 (74.7–91.4)	0.23	0.41	0.18
FEF_25–75%_ (L/s)	1.0 (0.6–1.6)	1.1 (0.6–1.8)	1.0 (0.7–1.6)	0.04	<0.01	0.05
FEF_25–75%_ (%pred)	34.0 (22.0–57.0)	41.0 (25.0–64.0)	37.8 (29.9–62.7)	0.04	0.88	0.05
BD chg (ml)	290.0(180.0–440.0)	160.0(40.0–340.0)	170.0(70.0–300.0)	<0.01	0.87	0.00
BD chg (%)	18.0 (11.0–28.0)	10.0 (2.0–22.0)	11.6 (5.0–20.7)	<0.01	0.10	0.00

Note: ^a^Wilcoxon and ^b^Friedman tests; *FVC* forced vital capacity, *FEV1* forced expiratory volume in 1 s, *FEF* forced expiratory flow, *BD* bronchodilator; data presented as median and interquartile range

A comparison between spirometries of V1 and V8, after the initial improvement has supposedly reached a plateau, shows that the FEV_1_ and FVC declined significantly both in absolute and percent of predicted values. FEV_1postBD_ did not change significantly between V0 and V1, but declined by -27.1 (−51.1–1.4) ml/yr between V1 and V8, using data of all patients with complete spirometry in V1 and V8.

## Discussion

Our sample comprised mostly adult subjects with previous long-standing untreated asthma, characterized by persistent symptoms, frequent exacerbations and low lung function. After 1 year of regular treatment with inhaled corticosteroids and LABA they had a clear reduction in exacerbations and improvement in lung function. From the end of the first year of treatment, which we took as a stable baseline to look at subsequent lung function decline, to the end of the study period, 7 years later, there was a further reduction in emergency room visits. However, this was associated with an increase in the dose of inhaled corticosteroids and the requirement for oral corticosteroids, based on a written action plan coupled with a patient education program. During the 7 years of subsequent regular treatment, we observed a PreBD decline in FVC, FEV_1_ and FEF_25–75%_ in absolute figures. FVC and FEV_1_ also declined in percent of predicted values. The PostBD observations, which are more appropriate to study lung function decline among subjects with obstructive lung diseases, confirmed a decline in the absolute figures for FVC, FEV_1_ and FEF_25–75%_. Again, there was also a reduction in the percent of predicted values for FVC and FEV_1_. The median decline of 184 PostBD FEV_1_ analyzed was −27.1 (−51.1–1.4) ml/yr between V1 and V8, which is not far from the average for healthy subjects in this age range reported from studies in other continents. It is remarkable that we did not observe any variation in the PostBD FEV_1_/FVC ratio during the entire period from the enrollment to the end of the study, which suggests it is the least responsive of the spirometric indexes of airway obstruction. A linear and unidirectional behavior of FEV_1_ and FVC during the follow-up, improving in the first year and decreasing subsequently, could be an alternative explanation for the relatively sustained FEV_1_/FVC ratio. Finally, it is noteworthy that a clear-cut reduction of FEV_1_ response to bronchodilator was detected after the first year of treatment and remained the same at the end. We didn’t find any significant association between change in FEV_1_ and response to bronchodilator. We speculate the reduction in bronchodilator response from V0 to V1 was likely related to the preBD FEV_1_ increase, with less room for dilatation. But this could not be an explanation for the change from V1 to V8. In this case, we consider the most likely explanation is a trend towards fixed airway obstruction.

Our study demonstrated that there is a small decline in lung function above the physiologic range despite of increasing dose of inhaled corticosteroids during 7 years of follow-up after 1 year of stabilization in regular treatment. This is the first estimate, to our knowledge, of decline in lung function among subjects with long-standing previously untreated severe asthma, now under regular and strictly monitored treatment for an extended follow-up period.

Various studies have demonstrated the benefits of inhaled corticosteroids to lung function in adults and children with mild and moderate asthma for periods of regular treatment up to 3 years [8–10, 12]. Early treatment of asthma seems to be necessary to obtain optimal benefits of inhaled corticosteroids in lung function [8], especially in reducing airway remodeling and preventing fixed airway obstruction [28, 29]. The long-term benefits of the regular use of inhaled corticosteroid and LABA combination for subjects with previously untreated severe asthma, including improvement in lung function, may surpass the 1 year we decided to adopt as the baseline plateau to look at the FEV_1_ postBD decline for pragmatic reasons. If this assumption is correct, our median measures of lung function decline express a balance between some additional improvement, which most certainly varies widely among subjects, followed by the true decline. If this speculation is correct the dimension of the decline is greater than we have measured, thus favouring our interpretation that there is indeed a decline above that expected, in spite the treatment with inhaled corticosteroids.

In our present study, there was an increased in the long-term utilization of inhaled and oral corticosteroids, upon medical guidance, with consequent reduced emergency visits. Nevertheless, we have seen a decline in lung function parameters after 7 years. Clinical improvement was probably related to reduction of bronchial hyper-responsiveness due to increasing dose of inhaled corticosteroids during follow-up. We did not measure bronchial hyper-responsiveness but we observed a decrease in the response to bronchodilator, which may be a proxy of hyper-responsiveness. A dissociation between symptoms control and lung function trends was observed in other studies [30].

Some limitations can be pinpointed in our study. First, we have no control group of subjects without asthma from the same specific population. Therefore, lung function decline was estimated on the basis of predicted Brazilian values not necessarily similar to our context of ethnicity mix, socioeconomic and health status. The majority of our cases were female, over 40 years of age and overweight. The predominance of women is expected because asthma is more frequent and severe in adult females as it was demonstrated in other studies [31, 32], but our proportion (82 %) was above those of previous reports from elsewhere. We suspect it might be influenced by a greater rate of unemployment, which increases availability for attending medical services among females, but we have no clear explanation for this observation yet. This certainly poses a limitation to the external validity of our findings. We have evaluated the annual change of FEV_1_ based in tests performed in V1 and V8. We found our FEV_1_ change calculation was acceptable, as it has been often used in the literature [13, 14, 33]. Nevertheless, we recognize this as a limitation of our study, as it would be better to have extra points to draw a more precise regression curve.

Although the absolute values of lung function have a near physiological decline over time, the percent of predicted values are not expected to decline because they are already adjusted for age. Therefore we face conflicting information: the absolute decline looks is not far from the normal, but there is a decline in the percent of predicted too. Standards of lung function normality have been established for the Brazilians, whereas studies on lung function decline were never done in the Brazilian population. Therefore, we consider our interpretation shall be that we have observed a mild decline over the average in lung function after 7 years, in spite of regular treatment including inhaled corticosteroids at high doses combined with LABA and early intervention with oral corticosteroids to halt exacerbations, according to a written action plan.

It is important to continue to follow up this cohort closely to see the trends as the subjects age. It is crucial also to dissect lung function decline on an individualized analysis, rather than looking at the median of the entire group. We intend to sort out which are the best means to look at clusters of subjects according to their rate of decline in lung function and search for determinants of a steeper decline.

## Conclusions

We conclude that currently available proper treatment with a combination of inhaled corticosteroids and LABA is not sufficient to prevent lung function decline, although it improves asthma control and reduces emergency visits, in subjects with previous untreated severe asthma from under privileged populations in Brazil.

## Abbreviations

ACQ: Asthma control questionnaire
AQLQ: Asthma quality of life questionnaire
BD: Bronchodilator
BMI: Body mass index
COPD: Chronic obstructive pulmonary disease
FEF: Forced expiratory flow
FEV_1_: Forced expiratory volume in first second
FEV_1_/FVC: Relation between Forced expiratory volume in first second and forced vital capacity
GINA: Global initiative for asthma
HF: Heart failure
ICU: Intensive care unit
LABA: Long acting beta 2 agonist
PEF: Peak expiratory flow
ProAR: Program for control of asthma in Bahia
SBPT: Brazilian thoracic society
SUS: National Health System
UFBA: Federal University of Bahia
